# Cu_4_SnS_4_-Functionalized Absorbent Pads-Derived Carbon as a Bifunctional Electrode for Supercapacitors and Hydrogen Evolution Reaction

**DOI:** 10.3390/nano16120773

**Published:** 2026-06-19

**Authors:** Romiyo Justinabraham, Arulappan Durairaj, John H. T. Luong, Samuel Vasanthkumar, Moorthy Maruthapandi

**Affiliations:** 1 Department of Chemical Sciences and the Radical Research Center, Ariel University, Ariel 40700, Israel; 2 Center of Materials and Nanotechnologies, Faculty of Chemical Technology, University of Pardubice, Nam. Cs. Legii 565, 53002 Pardubice, Czech Republic; 3 CATRIN—Regional Centre of Advanced Technologies and Materials, Palacký University, Slechtitelu 27, 77900 Olomouc, Czech Republic; 4 Bar-Ilan Institute for Nanotechnology and Advanced Materials, Bar-Ilan University, Ramat-Gan 52900, Israel; 5 Department of Chemistry, Bar-Ilan University, Ramat-Gan 52900, Israel; 6 School of Chemistry, University College Cork, T12 YN60 Cork, Ireland; 7 Department of Applied Chemistry, Karunya Institute of Technology and Sciences, Coimbatore 641114, India

**Keywords:** diaper waste, Cu_4_SnS_4_, energy storage, hydrogen evolution reaction

## Abstract

The conversion of bio-waste into functional energy materials provides a robust platform for addressing both environmental and energy challenges. In this paper, discarded absorbent pads are transformed into carbon-rich frameworks, which is followed by the fabrication of composites through the incorporation of Cu_4_SnS_4_ (CSS) for dual electrochemical applications. Integrating CSS into the waste-derived carbon matrix induces strong synergistic effects, improving electrical conductivity, increasing active-site availability, and accelerating charge-transfer kinetics. Comprehensive physicochemical analyses confirmed the successful formation of a well-integrated heterostructure composite with favorable structural and surface characteristics. Electrochemical evaluations further demonstrated that CSS-modified carbon exhibits superior bifunctional performance. In a two-electrode configuration, the composite delivers an energy density of 12.08 Wh kg^−1^ at a power density of 250 W kg^−1^ along with excellent cycling stability in supercapacitor applications. As an electrocatalyst, it achieves a low overpotential of 268 mV at −10 mA cm^−2^ and a small Tafel slope of 75 mV dec^−1^, reflecting efficient reaction kinetics. The strong durability observed in both systems underscores the structural integrity and long-term operational stability of the material. Overall, this paper advances a sustainable waste-to-resource strategy for fabricating multifunctional carbon-based composites, offering a promising platform for integrated energy-storage and hydrogen-generation technologies.

## 1. Introduction

The energy crisis is a major global problem in the 21st century due to overpopulation and industrialization. Currently, many energy sources, such as fossil fuels, nuclear power, and renewable energy sources, satisfy energy needs. Nuclear power and fossil fuels emit highly toxic gases (SO, CO, and NO) and radioactive wastes that are difficult to manage. The availability of fossil fuels is decreasing rapidly due to overconsumption at the same time. Therefore, the need for renewable and green energy sources is very important for current and future generations [1,2,3,4]. Researchers are currently focusing on finding alternative energy sources instead of nonrenewable and nuclear energy sources. In this viewpoint, hydrogen (H_2_) energy is the most advanced, safest, non-contaminant, and renewable energy source in current research. It has an excellent energy density (283 kJmol^−1^), and the by-product of the H_2_ generation is water (H_2_O). Hydrogen is produced by several electrolysis methods [5,6]. Among them, electrochemical water splitting has developed as the most cost-effective and highly promising approach for hydrogen generation. The electrocatalytic H_2_ evolution reaction includes two different mechanisms, such as hydrogen evolution reaction (HER) and oxygen evolution reaction (OER), that take place on both the cathode and anode simultaneously [7].

H_2_ ⟶ 2H^+^ + 2e^−^ (1)

H_2_O ⟶ O_2_ + 4H^+^ + 4e^−^ (2)

In addition, energy generation and storage are both crucial. During a natural disaster, using conventional energy sources to obtain electricity can be difficult. Therefore, the major problem is satisfying future energy demand using current energy sources. Therefore, developing new storage methods to keep the generated energy is essential. Many researchers are looking into developing energy storage devices. Among the numerous energy storage systems, supercapacitors are the most efficient energy storage devices due to their remarkable attributes, such as high-power density, rapid charge–discharge capabilities, long-lasting cycling stability, and ease of handling when compared to conventional capacitors and batteries [8,9]. Supercapacitors are widely used in various industries, including power source development, telecommunications equipment, industrial machinery manufacturing, and electric vehicle production [10,11]. Based on their charge-storage mechanisms, supercapacitors can be categorized into two primary groups: (i) Electric Double Layer Capacitors (EDLCs) and (ii) pseudocapacitors (Pc). In Pc, an electrochemical reaction occurs quickly and irreversibly on the electrode surface, whereas in EDLC, charge distribution occurs electrostatically at the electrode–electrolyte interaction [12].

Therefore, it is necessary to prepare low-cost materials with excellent efficiency for hydrogen generation and energy storage. Carbon-based materials have attracted much attention owing to their large specific surface area, excellent stability, enhanced catalytic loading, and outstanding electrical conductivity. Biochar has received more attention than other carbon-based materials because of its superior electrochemical properties, higher carbon content, low cost, and abundance of natural raw materials [13]. Several biological waste materials, including rice husk, pine cones, bamboo, fruit peels, and lotus stems, are transformed into carbon-rich biochar [14,15]. Sanitary-related materials, such as napkins and diapers, are crucial in human society due to their importance. Diapers are an important and unavoidable material for children, patients, and elderly people with 250 million diapers being used daily. Diaper wastes are considered as medical waste, and the biodegradation and decomposition of single-use disposable diapers might take up to 500 years. The resulting diaper waste poses a significant solid-waste management challenge due to its complex composition and high volume. Most used diapers are disposed of through landfilling or incineration, which are practices that contribute to landfill overcrowding, greenhouse-gas emissions, loss of valuable resources, and secondary pollution [16].

So the proper disposal of these enormous amounts of diaper waste, which pollute the ecosystem, is a significant challenge [17,18]. The conversion of used sanitary diapers into useful carbon material for various applications is an alternative way to handle this large quantity of waste. Transition metal dichalcogenides such as MoS_2_, MoSe_2_, WS_2_, NiS_2_, FeS_2_, CoSe_2_, and CoS_2_ are being developed as HER electrocatalysts. However, its potential is restricted by the low electrical conductivity, few active edges, and a negligible surface area [19,20,21,22]. Similarly, the discovery of highly efficient and cost-effective catalytic semiconductors from abundant raw sources is highly important to overcome these limitations. In recent times, abundant transition metal dichalcogenides have been discovered as highly promising catalysts for the HER. At present, there is a growing interest in ternary semiconductors, specifically Cu_4_SnS_4_ (CSS), which has emerged as a significant p-type semiconductor with a small or moderate band gap. CSS possesses several desirable characteristics, such as being cost-effective, environmentally friendly, and non-toxic. CSS has attracted considerable attention owing to its remarkable thermal, electrical, and optical properties, making it a promising candidate for photovoltaic devices, photocatalytic dye degradation and water splitting [23,24,25,26]. Moreover, CSS has attracted increasing attention for use in supercapacitors and as an electrocatalyst for the hydrogen evolution reaction (HER) owing to its favorable electrical conductivity, multiple redox-active metal centers, and sulfur-rich composition. Several studies have investigated Cu_4_SnS_4_ and related Cu–Sn sulfides as efficient electrode materials, demonstrating promising charge-storage capability and catalytic activity [27,28,29,30]. However, the performance of pristine Cu_4_SnS_4_ is often constrained by structural instability during long-term cycling, the limited exposure of active sites, and restricted electron-transport pathways. Despite recent advances, superior HER activity in Cu_4_SnS_4_-based systems is typically achieved only when the material is integrated with carbon-based frameworks [31].

In this paper, electrochemically active CSS was synthesized through a hydrothermal reaction, while sanitary diaper waste-derived biochar (Diachar) was obtained via a controlled carbonization process. Subsequently, the synthesized CSS was uniformly decorated onto the Diachar surface using a simple and efficient hydrothermal approach. The resulting CSS/Diachar composite exhibits several desirable properties, including low cost due to waste-derived carbon, good structural stability, and excellent recyclability. These features make it a promising candidate for hydrogen (H_2_) evolution through electrocatalytic water-splitting applications.

## 2. Materials and Methods

All the experimental procedures are in the Supplementary Information File.

## 3. Results and Discussion

### 3.1. X-Ray Diffraction (XRD) Analysis

**Figure 1:**
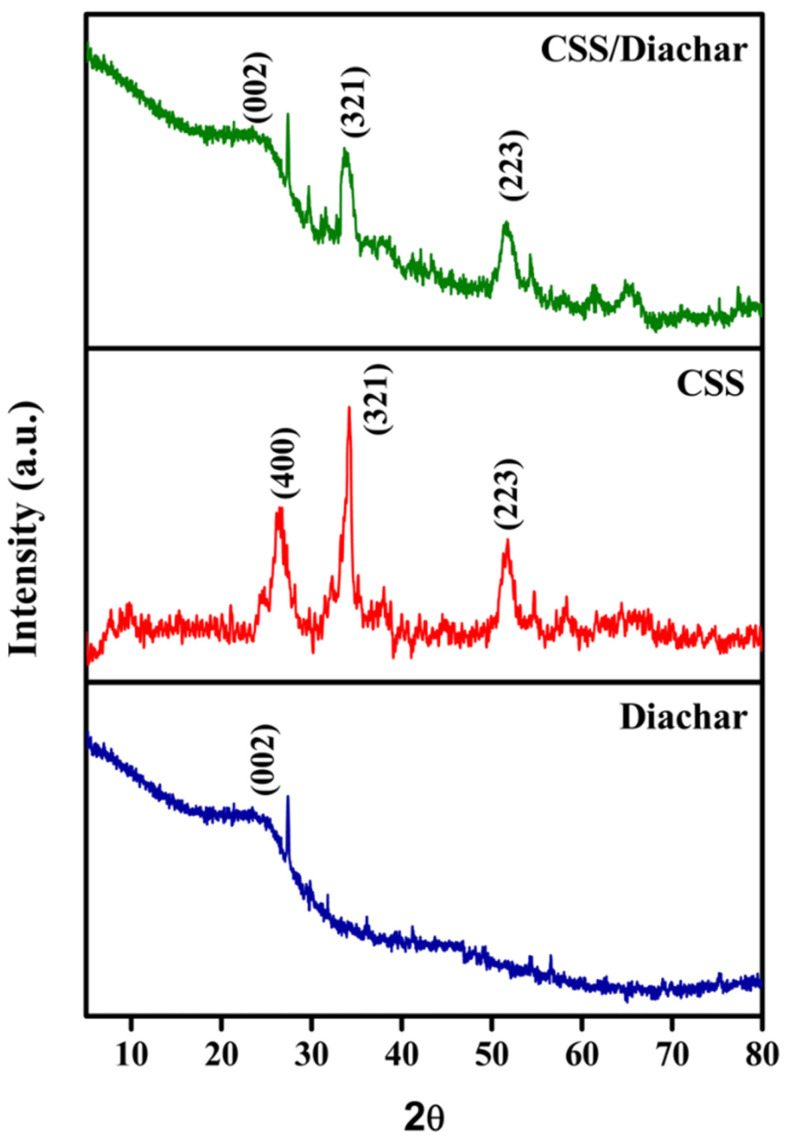
The XRD pattern of the prepared Diachar, CSS, and CSS/Diachar composite.

XRD analysis was used to study the crystal nature of the prepared materials. In Figure 1, the prepared Diachar shows the broad XRD peak at 25–30°, and the corresponding crystal plane is (002). The broad peak and the crystal plane (002) indicate the amorphous and graphitic nature of the prepared Diachar material [32,33]. The diffraction peak of CSS exhibits three different peaks at 26.4°, 34°, and 51.8°, and the corresponding crystal planes are (400), (321), and (223), respectively [JCPDS #029-0584] [34]. The CSS/Diachar composite shows all the diffraction peaks of the parent material. This finding reveals the excellent incorporation of CSS into the Diachar material, and the composite has the properties of the parent materials. The Debye–Scherrer equation was used to calculate the grain sizes of the synthesized materials. The calculated crystal size of the prepared CSS and CSS/Diachar was 10.8 nm and 6.11 nm, respectively. The incorporation of CSS into Diachar leads to a decrease in the grain size of the composite material.

### 3.2. Scanning Electron Microscopy Analysis

**Figure 2:**
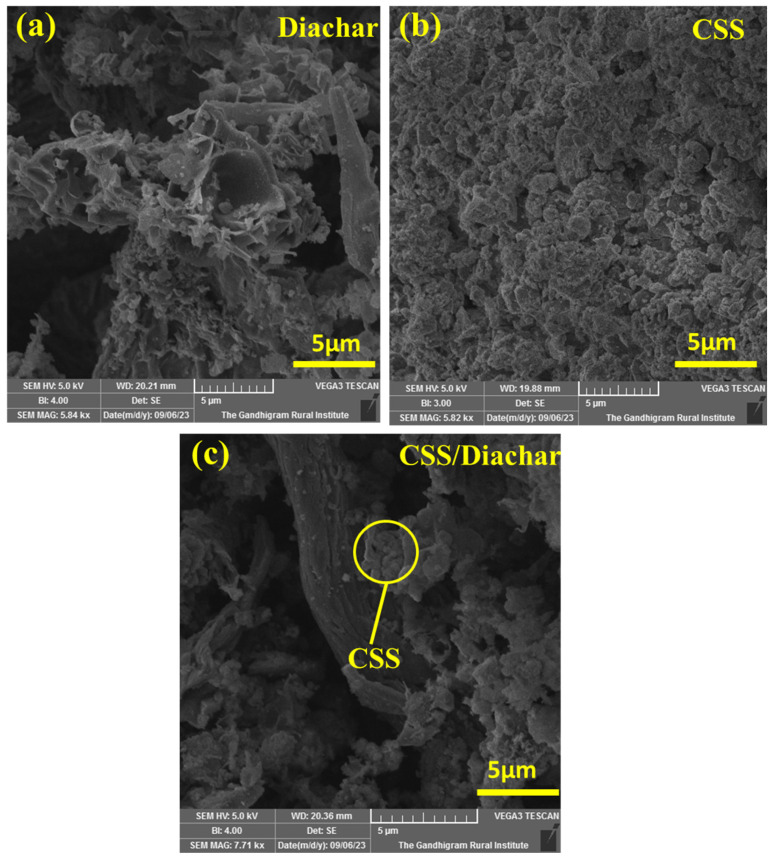
Scanning electron microscopy analysis of (**a**) Diachar, (**b**) CSS, and the (**c**) CSS/Diachar composite.

The surface morphology of the prepared materials was examined by SEM analysis. From this analysis, the prepared Diachar shows a sheet-shaped morphology (Figure 2a). In Figure 2b, the SEM analysis of CSS exhibits flake-shaped morphology. The SEM morphology of the CSS/Diachar composite (Figure 2c) shows that the CSS nanoflakes are excellently distributed on the surface of the Diachar and reveal the excellent incorporation of parent materials.

### 3.3. Elemental Analysis

**Figure 3:**
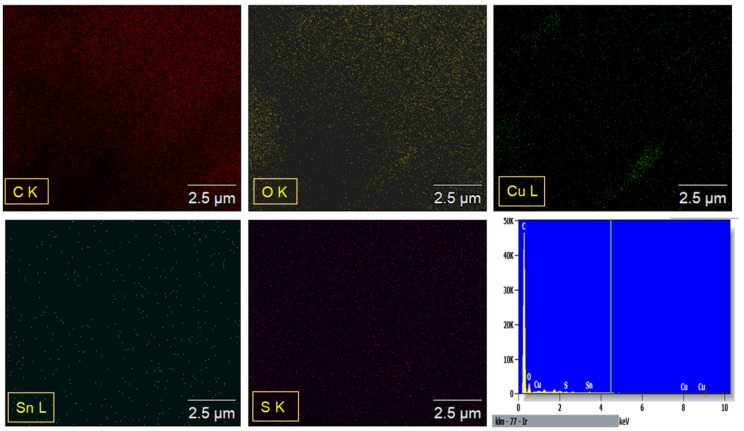
Elemental mapping and EDS analysis of the prepared CSS/Diachar composite, illustrating the spatial distribution and compositional presence of key elements (C, O, Cu, Sn, and S) within the composite material.

The elemental composition of the prepared material was analyzed using energy-dispersive X-ray analysis and mapping analysis (Figure 3). The prepared CSS/Diachar composite contains carbon (C), oxygen (O), copper (Cu), sulfur (S), and tin (Sn), and no other elements are present in the composite material. In sample CSS/Diachar, there is a homogenous distribution of Cu, Sn and S in a nearly stoichiometric ratio of 4:1:4 on a carbon moiety. The elemental compositions were determined to be carbon (72.33%), oxygen (19.51%), copper (3.81%), tin (1.31%), and sulfur (3.14%). The homogeneous distribution of Cu, Sn and S in the composite confirms the successful incorporation of Cu_4_SnS_4_ on the surface of Diachar.

### 3.4. Raman Spectroscopic Analysis

**Figure 4:**
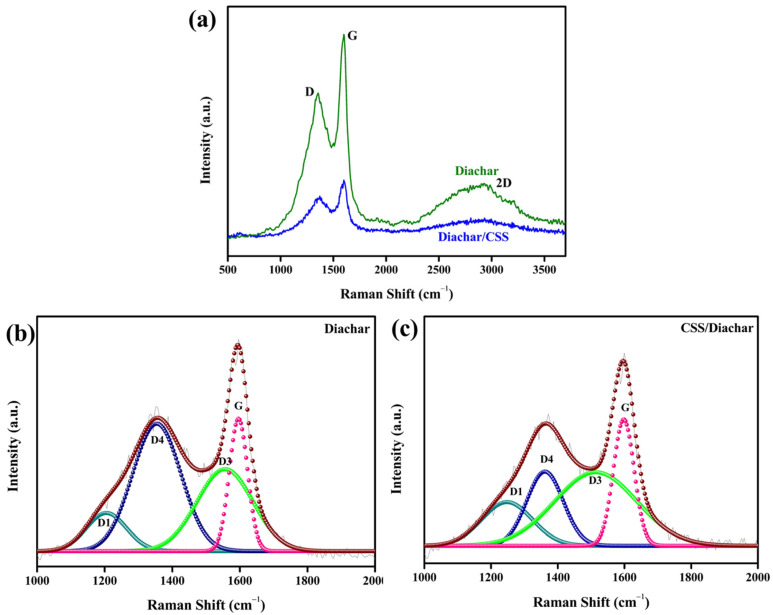
(**a**) Comparative Raman analysis of the prepared CSS/Diachar composite; deconvoluted Raman spectra of (**b**) Diachar and (**c**) the CSS/Diachar composite.

A Raman spectroscopic analyzer was used to examine the defects in the structure of the prepared CSS, Diachar, and CSS/Diachar composite (Figure 4). The Raman spectra of the Diachar and CSS/Diachar composite exhibit two sharp peaks at 1595 cm^−1^ and 1354 cm^−1^ corresponding to the G and D bands (Figure 4a) [35,36]. Moreover, due to the structural nature of graphene, the 2D band appeared at 2200–3400 cm^−1^ [37]. The calculated I_D_/I_G_ ratio for Diachar is 0.9214 from the D and G bands. In the CSS/Diachar composite, the I_D_/I_G_ ratio is increased by 0.973 due to the increased disordered/ordered graphitic structure. The Raman spectra of the prepared Diachar and the CSS/Diachar composite were deconvoluted into four peaks using the Lorentz and Gaussian method (Figure 4b,c).

The deconvolution images of the Diachar and composite show the G band at 1594 cm^−1^ and 1597 cm^−1^, respectively, and these G bands correspond to the E_2_g symmetry in the graphite layers. In both the Diachar and the composite, the D1 bands at 1204 cm^−1^ and 1245 cm^−1^ were revealed as aromatic clusters with more than six rings. The D3 and D4 peaks of Diachar are exhibited at 1553 cm^−1^ and 1354 cm^−1^, respectively. The deconvoluted spectra of the CSS/Diachar composite show D3 and D4 peaks at 1511 cm^−1^ and 1362 cm^−1^, respectively [33]. This analysis shows that the CSS was excellently incorporated with the surface of the Diachar and reveals the presence of graphitic carbon. Furthermore, the Raman spectra of both Diachar and CSS/Diachar were similar, revealing that the structure of Diachar was intact even after the CSS incorporation.

## 4. Electrochemical Investigations

### 4.1. Electrocatalytic Hydrogen Evolution Reaction (HER)

**Figure 5:**
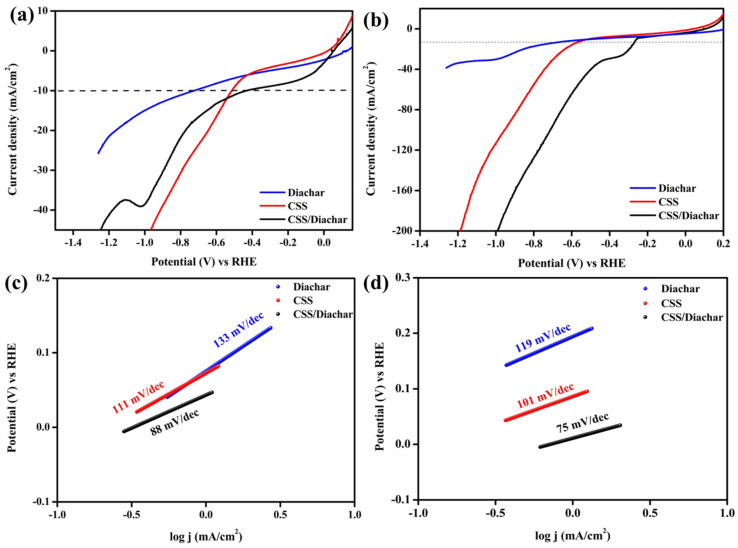
Linear sweep voltammograms of Diachar, CSS, and CSS/Diachar at (**a**) basic medium and (**b**) acidic medium. A Tafel slope of the prepared Diachar, CSS, and CSS/Diachar at (**c**) basic medium and (**d**) acidic medium for HER.

The electrocatalytic hydrogen evolution reaction activity of the prepared Diachar, CSS, and CSS/Diachar was determined using various techniques, including Tafel slope, overpotential, electrochemical impedance analysis, and stability. The HER activity of the prepared materials was evaluated in both acidic and alkaline media. The HER activity of the prepared Diachar, CSS, and the CSS/Diachar was analyzed using a drop-casting modified graphite sheet. Figure 5a shows the LSV curves of the prepared CSS and CSS/Diachar. The raw CSS and Diachar show slightly poor HER activity, and the composite exhibits enhanced electrocatalytic activity. In addition, the CSS/Diachar composite shows a minimum overpotential compared with the parent materials. The calculated overpotentials in the 0.5 M KOH medium were 720, 510, and 412 mV for Diachar, CSS, and CSS/Diachar, respectively, at a current density of 10 mA/cm^2^. Meanwhile, the HER activity of the materials was studied in an acidic medium electrolyte (Figure 5b). At 0.5 M H_2_SO_4_ electrolyte solution, the calculated overpotential from the LSV curves was 680, 560, and 268 mV for Diachar, CSS, and CSS/Diachar, respectively, at a current density of 10 mA/cm^2^. The acidic electrolyte medium shows excellent HER activity compared with the basic electrolyte medium. The Tafel slopes were used to determine the HER activity of the prepared materials. The Tafel slope can be used to determine the HER mechanism of various catalysts. In the basic medium, the calculated Tafel slopes of the prepared Diachar, CSS, and CSS/Diachar were 133 mV/dec, 111 mV/dec, and 88 mV/dec, respectively (Figure 5c). However, in the presence of an acidic electrolyte, the calculated Tafel slopes of Diachar, CSS, and CSS/Diachar were 119, 101, and 75 mV/dec, respectively (Figure 5d). From these findings, the prepared CSS/Diachar composite shows an excellent Tafel slope value compared with the parent material and reveals the Volmer–Heyrovsky mechanism in an acidic medium. The enhanced electrocatalytic performance of the CSS/Diachar composite can be attributed to the strong synergistic interaction between the Cu–Sn–S (CSS) nanostructure and the conductive Diachar matrix. The incorporation of Diachar provides a high surface area and conductive network, which facilitates rapid electron transport and reduces charge transfer resistance during the HER process. Simultaneously, the uniform dispersion of CSS on the Diachar surface increases the exposure of catalytically active sites and prevents particle agglomeration, thereby improving catalytic efficiency. The reduced overpotential and smaller Tafel slope values of the composite compared to the individual components indicate accelerated reaction kinetics. In acidic media, the Tafel slope (~75 mV/dec) suggests that the HER follows a Volmer–Heyrovsky mechanism, where the electrochemical desorption step is rate limiting. Moreover, the improved performance in acidic electrolyte compared to alkaline conditions can be attributed to the higher availability of protons and more favorable adsorption–desorption dynamics. Overall, the synergistic coupling between CSS and Diachar enhances conductivity, active site accessibility, and reaction kinetics, leading to superior HER activity. The recently reported Cu–Sn–S and carbon-based materials for hydrogen evolution reaction applications are shown in Table S1 [38,39,40,41,42,43,44,45].

**Figure 6:**
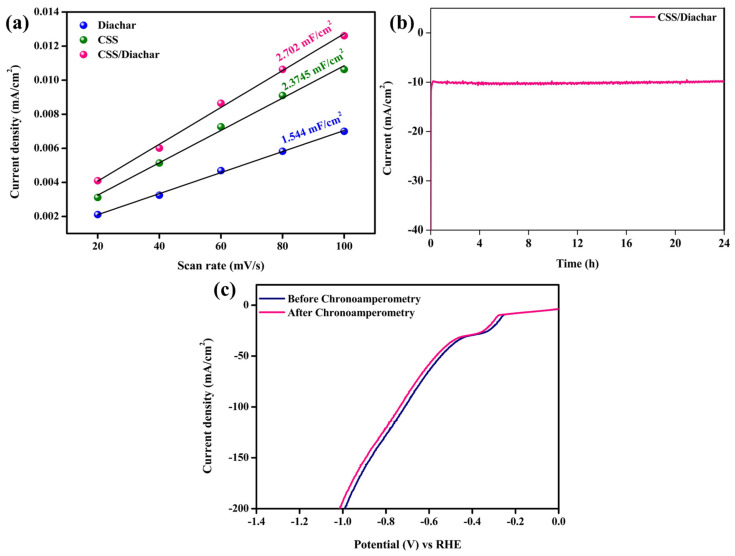
(**a**) Estimated electrochemically active surface area (ECSA) of Diachar, CSS, and CSS/Diachar measured in the potential range of 0–0.1 V vs. RHE; (**b**) chronoamperometry curve of CSS/Diachar at 268 mV vs. RHE. (**c**) LSV curves recorded before and after chronoamperometry.

Additionally, the HER performance of the prepared electrocatalyst was calculated by the electrochemically active surface area (ECSA) method. The ECSA calculation is used to calculate the double-layer capacitance from the cyclic voltammetry (CV) curves with various scan rates in the potential range of 0–0.1 V vs. RHE. A linear correlation was observed between the positive and negative current densities in the middle of the scan range and the scan rate. The plot of current density versus scan rate provides a slope that has been used to compute the double-layer capacitance (Cdl). The graph is displayed in Figure 6a. ECSA was calculated using the formula ECSA = Cdl/Cs, where Cs is the electrode capacitance, given the Cdl value. The calculated ECSA is 2.702 mF/cm^2^ from the ECSA slope, which is higher than the Diachar (1.544 mF/cm^2^) and CSS (2.3745 mF/cm^2^). The stability of the electrocatalyst was examined using chromoamperometry measurements of the CSS/Diachar composite (Figure 6b). After 15 h of testing at 268 mV vs. RHE, it was revealed that the composite material exhibited good stability.

### 4.2. Supercapacitor Application

#### 4.2.1. Three-Electrode System

Initially, the electrochemical performance of the prepared CSS, Diachar, and CSS/Diachar was studied by various electrochemical techniques such as cyclic voltammetry (CV), galvanostatic charge–discharge (GCD) studies, and electrochemical impedance spectrum (EIS) using a three-electrode system.

**Figure 7:**
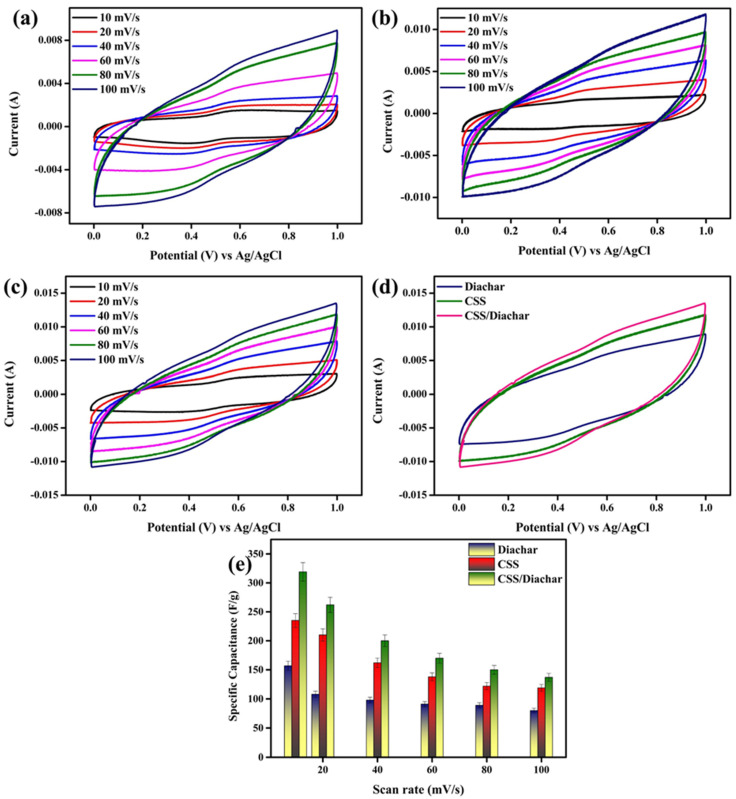
Cyclic voltammetry (CV) curves of (**a**) Diachar, (**b**) CSS, and (**c**) CSS/Diachar recorded in the potential range of 0 to 1 V at different scan rates, (**d**) comparison of the Diachar, CSS, and CSS/Diachar composites at a scan rate of 100 mV/s, and (**e**) estimated specific capacitance of Diachar, CSS, and CSS/Diachar composites at various scan rates.

The quasi-rectangular peak of the CSS, Diachar, and CSS/Diachar stay constant at different scan rates, as shown in (Figure 7a–d); the area of the CV curves varies with the scan rate (10 mV/s to 100 mV/s) due to the fast ion diffusion in the electrode material. The specific capacitance is increased by the efficient facilitation of electronic transport on the Diachar due to the incorporation of CSS. Equation (3) was used to determine the specific capacitance of the materials [46].

(3) $$C_{S}=\frac{\left(\int IdV\right)}{\left(s\times m\times \Delta V\right)}$$

Here, the specific capacitance (F/g), current (mA), scan rate (mV/s), mass of the active material (g), and potential window (V) are represented by the variables Cs, I, s, m, and ΔV, respectively. Using Equation (3), the specific capacitance of the synthesized Diachar, CSS, and CSS/Diachar are 157, 235, and 319 F/g, respectively, at 10 mV/s (Figure 7e). Furthermore, all of the prepared materials have higher specific capacitances and scan rates of 10 mV/s. The superior specific capacitance of the CSS/Diachar composite arises from the synergistic interplay between the pseudocapacitive behavior of CSS and the electric double-layer capacitance of the porous Diachar. The Diachar framework provides a conductive pathway that facilitates rapid electron transport, while CSS contributes additional Faradaic redox-active sites. Moreover, the intimate interfacial contact between CSS and Diachar enhances charge-transfer kinetics and improves the utilization of electroactive sites, resulting in markedly improved electrochemical performance compared with the individual CSS and Diachar components.

**Figure 8:**
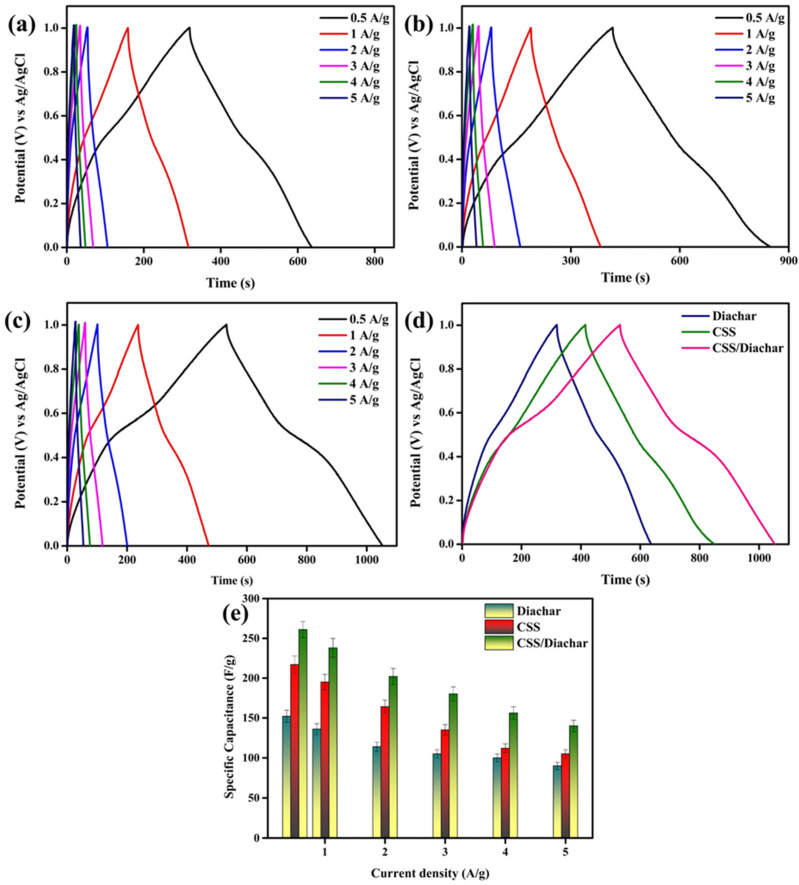
Galvanostatic charge–discharge (GCD) plot of (**a**) Diachar, (**b**) CSS, and (**c**) CSS/Diachar recorded in the potential range of 0 to 1 V at various current densities, (**d**) a comparison plot of Diachar, CSS, and the CSS/Diachar composite at a current density of 0.5 A/g, (**e**) comparison of specific capacitance of Diachar, CSS, and the CSS/Diachar composite at various current densities.

The specific capacitance and stability of the synthesized Diachar, CSS, and CSS/Diachar composite materials were determined by using the GCD method. Figure 8a–c show that the chronopotentiometric curves for all of the synthesized materials at different current densities of 0.5 A/g to 5 A/g in the potential window of 0 to 1 V. These GCD plots show that the discharge time of the prepared materials (Diachar, CSS, and CSS/Diachar composite) increased when the applied current was decreased, which resulted in a larger specific capacitance. Because of the faradic redox reaction, the charge–discharge plots of Diachar, CSS, and CSS/Diachar composite in all GCD plots exhibit nonlinear curves (Figure 8d).

The specific capacitance of the synthesized CSS/Diachar composite is higher than that of the parent materials. The specific capacitance of the prepared Diachar, CSS, and CSS/Diachar materials was determined using Equation (3)

(4) $$C_{S}=\frac{I\times \Delta t}{m\Delta V}$$

Here, I is the current (A), Δt is the discharging time (s), m is the weight of the active material (g), and ΔV is the potential window (V).

The specific capacitance of Diachar, CSS, and the CSS/Diachar composite at current densities of 0.5 A/g was determined to be 152, 217, and 261 F/g, respectively. This shows that the composite has superior specific capacitance compared with Diachar and CSS, respectively (Figure 8e). The improved capacitance behavior of the CSS/Dichar composite is attributed to the increased electron conduction between the electrolytes.

**Figure 9:**
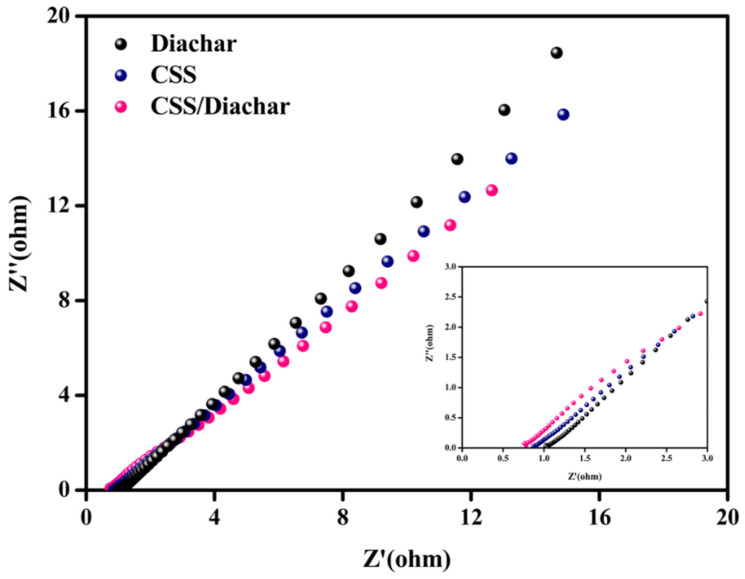
Electrochemical impedance spectroscopy (EIS) analysis plot of Diachar, CSS, and the CSS/Diachar composite.

The Nyquist plots of the CSS, Diachar, and CSS/Diachar composite were examined using EIS analysis. A three-electrode system with a frequency range of 0.1 Hz to 100 kHz was used for the EIS investigation (Figure 9). The Nyquist plots of these electrode materials show high and low-frequency regions. The high-frequency region exhibits a non-zero intercept with a negligible semicircle, suggesting low internal resistance and low charge transport properties, which leads to good conductivity and low resistivity. The calculated R_s_ values from the EIS analysis were 1.017, 0.8665, and 0.7712 Ω for Diachar, CSS, and CSS/Diachar, respectively. However, a 45–angled straight line in the low-frequency region (the Warburg resistance) reveals the outstanding capacitance performance of the synthesized CSS/Diachar electrode material. The electrolyte ion diffusion resistance into the bulk electrode is associated with the straight line length in the lower-frequency region. The shorter Warburg line indicates the lower impedance to electrolyte ion diffusion, which leads to good charge storage performance. From this EIS analysis, the prepared CSS/Diachar electrode material has a minimal solution and charge transfer resistance compared to Diachar and CSS electrode materials.

#### 4.2.2. Two-Electrode System

**Figure 10:**
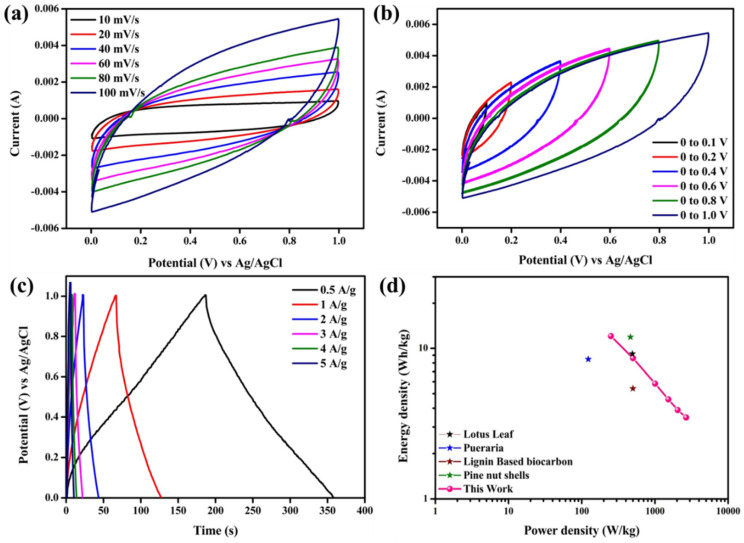
Electrochemical performance of the asymmetric supercapacitor in 0.5 M H_2_SO_4_ electrolyte: (**a**) CV plot recorded in the constant potential windows of 0 V to 1 V at various scan rates, (**b**) CV curves of the asymmetric supercapacitor recorded in the constant current of 100 mV/s at various potential windows, (**c**) GCD profiles recorded in the potential range of 0 V to 1 V at various current densities, (**d**) Ragone plot of the asymmetric supercapacitor in H_2_SO_4_ electrolyte solution.

CSS/Diachar (CD) exhibits excellent capacitive performance in the three-electrode system described above, which inspired the construction of the asymmetric two-electrode system. In this asymmetric two-electrode system, the CSS/Diachar and Diachar were used as positive and negative electrodes, respectively, and 0.5 M H_2_SO_4_ was used as an electrolyte. In CV studies, a detailed examination of the assembled asymmetric CD//Diachar supercapacitor was recorded at various scan rates between 10 and 100 mV/s within the potential window of 0 V to 1 V (Figure 10a).

In this paper, the area of the CV curves increased with increasing current, indicating optimal electrochemical capacitive behavior as well as fast ion diffusion. Moreover, the CV studies of the prepared CD//Diachar supercapacitor were analyzed at various potential windows from 0.1 to 1 V with a constant current of 100 mV/s (Figure 10b). The excellent capacitive behavior and good reversibility of the CV curves allow the rectangular shape to be retained from the lower to the higher working potential window. Figure 10c shows the GCD curves of the CD//Dichar asymmetric cell at different current densities, ranging from 0.5 to 5 A/g. At 0.5 A/g current density, the asymmetric CD//Diachar supercapacitor reaches a maximum capacitance of 87 F/g. The results provide a higher energy density of 12.08 Wh/kg with a power density of 250 W/kg, indicating a rapid ion/electron diffusion and remarkable energy and power densities. Figure 10d displays the Ragon plot of an asymmetric cell.

**Figure 11:**
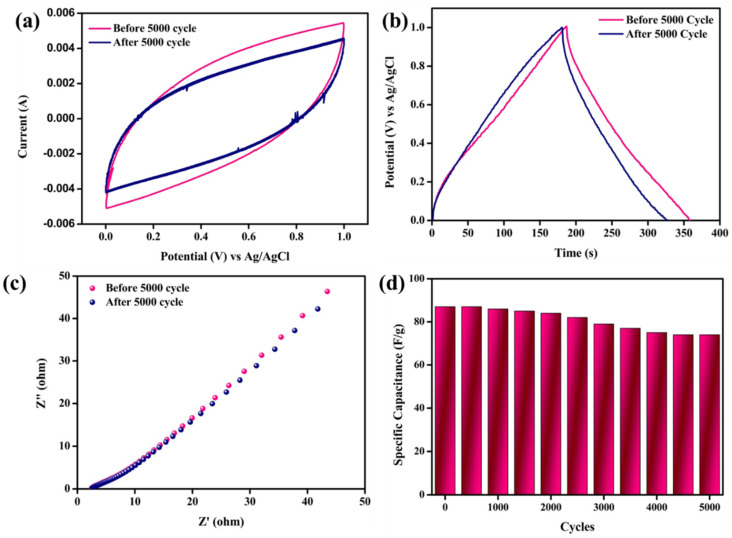
Stability performance of asymmetric supercapacitor recorded in the potential range of 0 V to 1 V at a specific current of 0.5 A/g: comparison of (**a**) CV and (**b**) GCD of before and after 5000 cycle performance, (**c**) comparison of EIS before and after 5000 cycle performance, (**d**) multi-cycle GCD performance plot of the asymmetric supercapacitor recorded in the potential range of 0 V to 1 V at 0.5 A/g current density.

The stability of the CD//Diachar asymmetric supercapacitor was studied in various studies, including CV, GCD, and EIS (Figure 11a–d). From these studies, the CD//Diachar asymmetric cell showed good cyclic stability during 5000 GCD cycles at 0.5 A/g current density with a specific capacitance retention of 85% of the initial capacitance.

## 5. Conclusions

A straightforward carbonization strategy was employed to convert disposable diaper-derived biological waste into a carbon-rich framework. The incorporation of Cu_4_SnS_4_ (CSS) markedly enhanced the electrochemical activity of the resulting carbon matrix. The synthesized materials were comprehensively characterized using XRD, SEM, EDX, and Raman spectroscopy, confirming successful composite formation and favorable structural features. The developed composite functions as a multifunctional catalyst, exhibiting strong performance in both electrochemical energy-storage and energy-conversion applications. In two-electrode asymmetric supercapacitor measurements, the material delivered a specific capacitance of 87 F g^−1^ at 0.5 A g^−1^ and retained 85% of its initial capacitance after 5000 charge–discharge cycles, demonstrating excellent cycling stability. It also achieved an energy density of 12.08 Wh kg^−1^ at a power density of 250 W kg^−1^. For hydrogen evolution reaction (HER) studies, the composite outperformed its individual parent components, achieving an overpotential of 268 mV at −10 mA cm^−2^ and a Tafel slope of 75 mV dec^−1^. Chronoamperometric analysis further confirmed its robust electrocatalytic durability, maintaining stable performance for up to 15 h. Overall, this paper establishes a sustainable waste-to-resource pathway for fabricating multifunctional carbon-based composites, offering a promising platform for integrated supercapacitor and hydrogen-generation technologies.

## Data Availability

The original contributions presented in this study are included in the article/Supplementary Material. Further inquiries can be directed to the corresponding authors.

## Supplementary Materials

The following supporting information can be downloaded at https://www.mdpi.com/article/10.3390/nano16120773/s1, Section 2: Materials and methods. Table S1: The recently reported Cu-Sn-S & carbon-based materials for hydrogen evolution reaction applications.
