# Supplementary material for: Cu4SnS4-Functionalized Absorbent Pads-Derived Carbon as a Bifunctional Electrode for Supercapacitors and Hydrogen Evolution Reaction
Source: Nanomaterials (Basel). 2026 Jun 19;16(12):773. doi: 10.3390/nano16120773 (PMC13305544; doi:10.3390/nano16120773)
Supplement: Supplementary file 1 [file nanomaterials-16-00773-s001.zip › nanomaterials-4379184-supplementary.pdf]

Supplementary Information

Cu_4SnS_4-Functionalized Absorbent Pads-Derived Carbon as a Bifunctional Electrode for Supercapacitors and Hydrogen Evolution Reaction

Romiyo Justinabraham[1,2], Arulappan Durairaj[3,4,5], John H.T. Luong[6], Samuel Vasanthkumar[7*] and Moorthy Maruthapandi[4,5*]

[1] Department of Chemical Sciences and the Radical Research Center, Ariel University, Ariel 40700, Israel; justinab07@gmail.com

[2] Center of Materials and Nanotechnologies, Faculty of Chemical Technology, University of Pardubice, Nam. Cs. Legii 565, 53002 Pardubice, Czech Republic

[3] CATRIN—Regional Centre of Advanced Technologies and Materials, Palacký University, Slechtitelu 27, 77900, Olomouc, Czech Republic; chemdraj@gmail.com

[4] Bar-Ilan Institute for Nanotechnology and Advanced Materials, Bar-Ilan University, Ramat-Gan 52900, Israel

[5] Department of Chemistry, Bar-Ilan University, Ramat-Gan 52900, Israel

[6] School of Chemistry, University College Cork, T12 YN60 Cork, Ireland; luongprof@gmail.com

[7] Department of Applied Chemistry, Karunya Institute of Technology and Sciences, Coimbatore 641114, India

* Correspondence: kumar2359@yahoo.com (S.V.); maruthapandimartin.m@gmail.com (M.M.)

2. Materials and methods

2.1 Materials:

Copper chloride dihydrate ($CuCl_2$. $2H_2O$), stannous chloride ($SnCl_2$), thiourea (CH_4N_2S), and N-methyl-2-pyrrolidone (NMP) were purchased from Merck, India, whereas polyvinylidene fluoride (PVDF) was purchased from Sigma Aldrich, India. Waste diapers were collected from nearby households around KITS, India. Double-distilled water (DDW) was prepared using double-distilled (DD) unit in our laboratory. A graphite sheet with 0.5 mm thickness was used as the working electrode.

2.2 Synthesis of Cu_4SnS_4 (CSS)

Typically, 4 mM of $CuCl_2$ was dissolved in 20 mL of water. Then, 1 mM of $SnCl_2$ and 4 mM of CH_4N_2S were added, and the mixture was stirred for 60 min. Subsequently, the prepared mixture was transferred to a stainless-steel Teflon-lined autoclave, and the reaction was carried out at 180 ^{o}C for 24 h. Then, the reaction setup was allowed to cool to room temperature. The precipitate was collected and washed several times using DDW, followed by EtOH, and dried in an oven at 80°C for 12 h.

2.3 Preparation of Diaper waste carbon (Diachar)

Briefly, the used diapers were dried in the sunlight for 2 days. The dried diaper waste was further cut into small pieces. These small pieces were carbonized at 450 ^{o}C for 4 h in a tube furnace under N_2 atmosphere. The obtained carbon was washed several times with DDW, followed by drying in an oven at 80°C for 12 h. The prepared diaper waste carbon was labelled as Diachar.

2.4 Preparation of CSS/Diachar

The prepared CSS and Diachar were mixed at a ratio of 20% and 80%, respectively. A higher Diachar content enhances electron transport and improves electrolyte accessibility within

the composite. To this mixture, 50 mL of EtOH was added, and the suspension was stirred for 30 min. The reaction mixture was then subjected to a hydrothermal treatment at 150 °C for 12 h. These conditions promote effective interaction and uniform deposition of CSS onto the Diachar surface, resulting in a stable composite structure. After the reaction, the product was washed thoroughly with deionized water and EtOH, followed by drying in an oven at 80 °C for 12 h.

2.5 Instrumentations

The physical and chemical nature of the prepared Diachar, CSS, and composite were examined using X-ray diffraction (XRD), scanning electron microscopy (SEM), energy dispersive X-beam spectroscopy (EDS) and Raman spectroscopy. The XRD spectrum was examined using an X-Shimadzu analyzer. while the confocal Raman spectrometer (Germany) was used to study the Raman spectra. The EDS analyzer-associated SEM instrument (Model: JEM-2100, Japan) is used to examine the presence of elements and the morphology of the materials.

2.6 Electrochemical studies

2.6.1 Electrode Preparation:

The preparation of a modified working electrode involves several steps. The PVDF and the prepared materials (CSS, Diachar, and CSS/Diachar) were mixed at a ratio of 1:9. A few drops of NMP were added, and the mixture was bath-sonicated for 60 min to achieve uniform dispersion. The prepared slurry was coated on to the surface of the graphite sheet electrode (1 x 1 cm) surface with a thickness of 0.5 mm) using the drop-casting method. The coated electrode was allowed to dry overnight at 80°C overnight. The mass loading of the active material on the working electrode was approximately 1 mg, and the complete electrochemical HER activity was performed at room temperature.

2.6.2 Supercapacitor studies

The electrochemical performance of the prepared materials was evaluated on a CHI-660C electrochemical workstation. The supercapacitance studies were done by a three-electrode system where the platinum wire, Ag/AgCl, and modified graphite sheet were used as the counter, reference, and working electrodes respectively, The CSS/Diachar-modified and Diachar-modified electrodes were used as positive and negative electrodes, respectively, in the two-electrode system. H_2SO_4(0.5M) was used as the electrolyte solution for the electrochemical studies in both two-electrode and three-electrode systems. The specific capacitance values were normalized to the mass of active material (F/g), while the current densities used for GCD measurements (A/g) and cycling stability tests were recorded in the potential window from 0V to 1V at 0.5A/g current density.

2.6.3 HER studies:

The hydrogen evolution reaction (HER) studies were conducted using a three-electrode configuration setup. Platinum wire, Ag/AgCl, and modified graphite sheet were used as the counter, reference, and working electrodes, respectively. 0.5M H_2SO_4 and 0.5M KOH solutions were used as the acidic and basic electrolytes for HER studies. The overpotentials vs Ag/AgCl of all the materials were calculated at a current density of 10 mA/cm^2. The obtained potential values are converted into reversible hydrogen electrode (RHE) potentials using the following equation (1):

$$E_{RHE} = E_{Ag/AgCl} + 0.209\ V + 0.059V*pH \qquad \text{------------------------} \ (1)$$

Table S1: The recently reported Cu-Sn-S & carbon-based materials for hydrogen evolution reaction applications

S. No	Material	Electrolyte	Overpotential (mV vs. RHE)	Tafel slope (mV dec^{-1})	Ref
1	Cu_2SnS_3/rGO	1M KOH	287	113	[38]
2	Cu_2ZnSnS_4	0.5M H_2SO_4	319	155	[39]
3	Cu_2FeSnS_4	0.5M H_2SO_4	470	153	[40]
4	Cu_3FeS_4-N/rGO	0.5 M H_2SO_4	276	92	[41]
5	$CuCo_2O_4$ @ Sargassum tenerrimum derived carbon	0.5M H_2SO_4	383	100	[42]
6	Co/N,S-Shrimp shells biochar	0.5M H_2SO_4	460	107	[43]
7	Microwave reduction GO	1M KOH	365.4	141.3	[44]
8	activated nitrogen and sulfur co-doped graphene	0.5M H_2SO_4	309	120.1	[45]
9	Cu_4SnS_4/Diachar	0.5M KOH	412	88	This work
		0.5M H_2SO_4	268	75	This work
